# Preparation of Cellulose Nanocrystals from Jujube Cores by Fractional Purification

**DOI:** 10.3390/molecules27103236

**Published:** 2022-05-18

**Authors:** Xiaorui Wang, Hao Le, Yanmei Guo, Yunfeng Zhao, Xiaorong Deng, Jian Zhang, Lianfu Zhang

**Affiliations:** 1 School of Food Science and Technology, Shihezi University, Shihezi 832003, China; wangxiaorui0923@163.com (X.W.); 20192111005@stu.shzu.edu.cn (H.L.); sdlcgtgym@163.com (Y.G.); yunfeng@shzu.com.edu.cn (Y.Z.); dxr20099@163.com (X.D.); 2 School of Food Science and Technology, Jiangnan University, Wuxi 214122, China

**Keywords:** jujube cores, cellulose nanocrystals, characterizations, purification

## Abstract

Jujube cores are fiber-rich industrial waste. Dewaxing, alkali treatment, bleaching, and sulfuric acid hydrolysis were used to generate cellulose nanocrystals (CNCs) from the jujube cores in this study. The morphological, structural, crystallinity, and thermal properties of the fibers were investigated using FE-SEM, TEM, AFM, FT-IR, XRD, and TGA under various processes. CNCs’ zeta (ζ) potential and water contact angle (WAC) were also investigated. The findings demonstrate that non-fibrous components were effectively removed, and the fiber particles shrunk over time because of many activities. CNCs had a rod-like shape, with a length of 205.7 ± 52.4 nm and a 20.5 aspect ratio. The crystal structure of cellulose Iβ was preserved by the CNCs, and the crystallinity was 72.36%. The temperature of the fibers’ thermal degradation lowered during the operations, although CNCs still had outstanding thermal stability (>200 °C). Aside from the CNCs, the aqueous suspension of CNCs was slightly agglomerated; thus, the zeta (ζ) potential of the CNCs’ suspension was −23.72 ± 1.7 mV, and the powder had high hydrophilicity. This research will be valuable to individuals who want to explore the possibility for CNCs made of jujube cores.

## 1. Introduction

In the agri-food industry, a large amount of waste is generated during the processing and production of raw materials, such as peels, cores, leaves, and other inedible parts. The disposal of these wastes can cause some pollution and environmental problems [1,2]. Therefore, it is very important for local and national economic development to find a more reasonable way to utilize waste, to make value-added utilization of waste, and to reduce environmental pollution as much as possible [3]. Jujube (*Ziziphus jujuba* Mill) is an important cash crop in China, with an annual output of 7.46 million tons in 2019. It contains many bioactive compounds and can be used for consumption as fresh or processed in various red jujube products (such as dried jujube, jujube slices, drinks, jam, jujube powder, etc.) [4]. Jujubes should be de-pitted before processing, which will produce a large amount of industrial waste: jujube cores. Except for a small amount of the jujube cores which are used as animal feed, the rest are discarded as waste and this waste can become a huge environmental problem. Therefore, the best solution for this problem is to improve the comprehensive value of the jujube industry to find an economic and reasonable way to deal with jujube cores and realize the reuse of this waste [5,6].

Jujube cores are lignocellulosic materials containing phenols, polysaccharides, oil, and other components, of which cellulose, lignin, and hemicellulose are the main components [7,8]. At present, the utilization of jujube cores is mainly focused on the use of jujube cores as adsorbents for water purification [9], activated carbon [10], biofuels [11], and functional components such as polyphenol extraction [12,13]. However, there are few studies on the utilization of fiber components in jujube cores.

Cellulose is one of the most abundant natural biopolymers, which is composed of D-glucopyranosyl linked by a 1,4-β-Glycoside bond [5,14,15]. It is the main component of the plant cell wall, which is often associated with hemicellulose, lignin, and resin [16]. There are both crystalline and amorphous regions in the cellulose structure. The crystalline cellulose is very suitable as an enhancer for other polymer systems due to its excellent mechanical properties, thermal stability, biodegradability, and environmental friendliness [17,18]. Cellulose can be decomposed into nanocellulose after chemical or mechanical treatment. According to their sources and separation methods, they can be divided into bacterial nanocellulose (BNC), cellulose nanocrystals (CNCs), and cellulose nanofibers (CNFs) [19,20].

CNCs are a kind of nanorod material obtained by removing the amorphous part of cellulose through intense acid hydrolysis or a coupling mechanical treatment process [21]. There is a wide range of sources and cellulose has been successfully separated from some agricultural wastes, such as softwood [22], flax, soy hulls [23], sisal fibers [24], tea waste [14], walnut shells [25], potato peel waste [26], coffee husks [27], and lemon (Citrus limon) seeds [20]. Because of its excellent mechanical, optical, non-toxic, biodegradable, and renewable properties, it is more prominent in food packaging and application, which has attracted the extensive attention of researchers [16]. The CNCs can effectively stabilize the interface, which due to the characteristics of having an anisotropic shape, having a stable electrostatic charge, being both hydrophilic and lipophilic, and being easy to form a dense interface network, make the CNCs show advantages in a Pickering emulsion [28,29]. The dimension, charge, crystallinity, and wettability of cellulose can be defined as a colloidal state. The colloidal state affects its film-forming ability, emulsification/foaming ability, encapsulation, and other functions [30]. Therefore, a detailed study of the morphology, charge, crystallinity, and wettability of CNCs can provide clear insights into the use of CNCs.

Natural fibers act as reinforcers to improve the mechanical strength, optical properties, and antibacterial properties of materials. The abundant fibrous material in jujube cores makes them a competitive material for developing different types of fibers. At present, a small number of scholars have studied the extraction of cellulose from jujube cores [5,8,31]. Among them, Wahib et al. [32] studied two methods to extract CNCs, and compared the crystallinity of the extracted CNCs and the economic cost of the two extraction methods. However, there are limited studies on the more specific structure and morphology of CNCs. The structure and morphology of polymers affect their properties. In this study, CNCs were obtained by hydrolyzing cellulose in jujube cores with sulfuric acid, and the functional group information contained in them was determined by FT-IR. The morphology and surface structure properties were determined by using FE-SEM, TEM, and AFM. The crystal structure and thermodynamic stability were determined by TGA. This study may provide useful insights into the efficient utilization of jujube cores by exploring the morphology, charge, crystallinity, and wettability of CNCs.

## 2. Results

### 2.1. Chemical Composition and Purification

The analysis of the initial composition of the jujube core powder was helpful to determine the extent of the subsequent chemical treatment process. The results showed that the jujube core powder contained a large amount of holocellulose (64.55 ± 4.56%), lignin (26.27 ± 3.74%), a small amount of Soxhlet extract (2.14 ± 0.21%), and ash content (0.38 ± 0.04%). The holocellulose included α-cellulose (40.48 ± 1.37%) and hemicellulose (24.07 ± 1.03%). As a result, jujube cores have a high cellulose content and can be employed as the primary raw material for cellulose extraction. After dewaxing, alkali treatment, and bleaching, the resulting product had a higher cellulose content and lower hemicellulose and lignin content: α-cellulose (80.47 ± 2.74%), hemicellulose (8.04 ± 1.21%), and lignin (0.57%). This indicated that after the purification treatment, lignin and hemicellulose were significantly removed and the resulting cellulose had a higher α-cellulose content, which was more suitable for the extraction of CNCs.

### 2.2. FT-IR Spectroscopic Analysis

**Figure 1:**
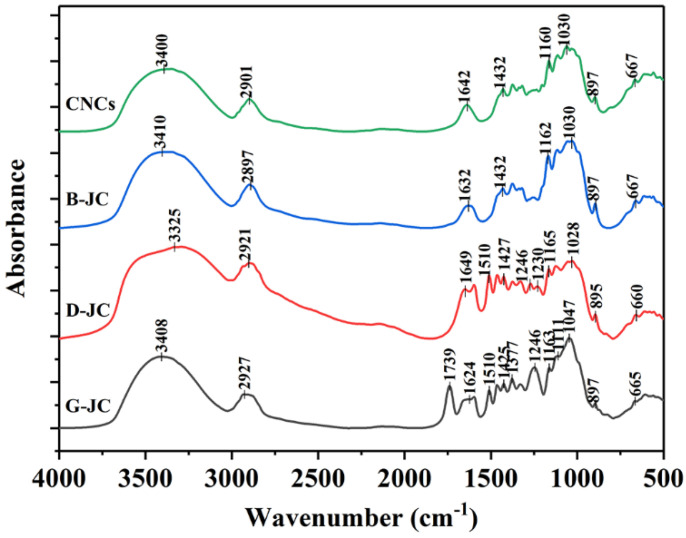
FT-IR spectra of G-JC, D-JC, B-JC and CNCs.

The efficiency of the separation and purification process can be analyzed further by FT-IR analysis of the jujube core powder (G-JC), the delignified jujube core (D-JC), the bleached jujube core (B-JC), and the CNCs. Figure 1 shows the FT-IR spectrum of the G-JC, D-JC, B-JC, and CNCs in the wavenumber range of 400–4000 cm^−1^. The main bands in the FT-IR spectra for the G-JC, D-JC, B-JC, and CNCs are listed in Table 1. Figure 1 clearly shows that G-JC contains lignin, hemicellulose, and other components. The peak located at 1739 cm^−1^ was due to the symmetric stretching vibration in the CC-’s in-plane of the aromatic ring in the lignin, and the carbonyl C=O stretching vibration in the lignin carboxylic acid bond or ester group in hemicellulose [33,34]. The peak at 1739 cm^−1^ disappeared in the D-JC’s spectrum, indicating that the NaOH treatment effectively removed lignin and hemicellulose [35]. Due to the presence of lignin and hemicellulose in G-JC and D-JC, the peaks are observed in the spectrum at wavelengths of 1246 cm^−1^, 1230 cm^−1^, and 1510 cm^−1^ [5,36]. On the contrary, these peaks disappeared in the B-JC’s and CNCs’ spectrums. It showed that after the separation and purification of the red jujube core powder, hemicellulose and lignin were effectively removed, and chemically purified cellulose was obtained.

In Figure 1, all samples showed a broad and strong absorption peak near 3400 cm^−1^ and a weaker absorption peak near 2900 cm^−1^, which were due to the stretch vibrations of O–H and C–H groups in the cellulose molecule [37,38]. The peak at 1640 cm^−1^ was related to the O–H bending vibration caused by the strong adsorption of cellulose and water. The peaks at 1047 cm^−1^ and 1030 cm^−1^ represent the vibration of the C–O–C pyranose ring in cellulose, and the peak near 897 cm^−1^ is related to the C–H swing in the β-glycosidic linkages of the glucose ring in the cellulose molecule [39]. Usually, the two peaks are related to the cellulose content, and the increase in the peaks represents the increase in the cellulose content [35,40]. It can be observed in the figure that as the separation and purification process progresses, the two peak intensities increase, indicating that as the separation and purification process progresses, the relative content of cellulose in the sample increases and the sample is further purified. The FT-IR spectra of the B-JC and CNCs are similar, indicating that CNCs maintain a structure consistent with cellulose molecules and that sulfuric acid hydrolysis does not destroy the chemical structure of cellulose.

**Table 1:** The main bands of FT-IR spectra for G-JC, D-JC, B-JC and CNCs.

Band Range (cm^−1^)				Changes in Functional Components	References
G-JC	D-JC	B-JC	CNCs		
3408	3325	3410	3400	Stretch vibration of O–H group in the cellulose molecule	[35,37,38]
2927	2921	2897	2901	Stretch vibration of C–H group in the cellulose molecule and asymmetry stretch vibration of CNCs	[5,35,36,39]
1739	-	-	-	Stretch vibration in the CC-’s in-plane of the aromatic ring in lignin, and the C=O vibration in the carboxylic acid bond of lignin or the carbonyl group in the ester group in hemicellulose	[33,34,35,39]
1624	1649	1632	1642	O–H bending vibrations of strong interaction between cellulose and water	[35,39,41]
1510	1510	-	-	C=C vibration of lignin	[5,36,39]
1425	1427	1432	1432	C–H vibrations in cellulose	[39,41]
1246	1246	-	-	C–O–C stretching vibration of acetyl linkage in lignin, hemicelluloses	[5,36,39]
1163	1165	1162	1160	C–O–C stretching vibration of β-glycosidic linkages between glucose units in cellulose	[38,39,42]
1047	1028	1030	1030	C–O–C pyranose ring in cellulose	[5,39,41]
897	895	897	897	C–H swing in the β-glycosidic linkages of glucose ring	[5,35,39,40]
665	660	667	667		

### 2.3. X-ray Diffraction Analysis

**Figure 2:**
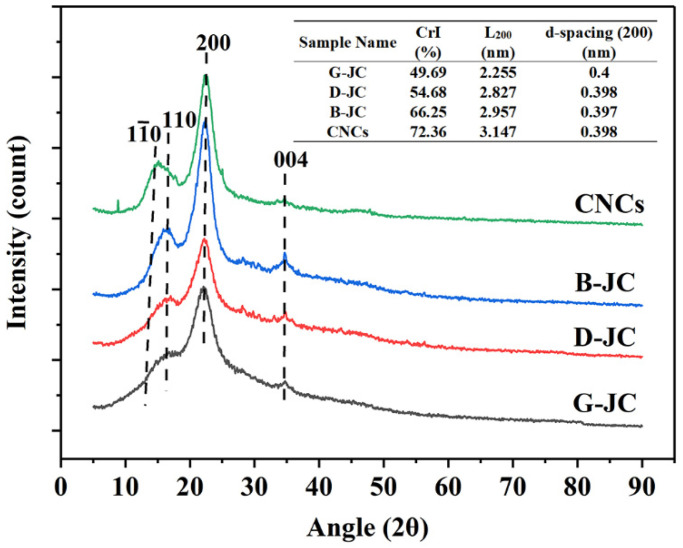
X-ray diffraction patterns and crystallinity index (*CrI*), crystallite size (L), and *d*-spacing of G-JC, D-JC, B-JC, and CNCs.

X-ray diffraction can evaluate the crystallization behavior of jujube core powder after different treatments and further judge the effect of chemical purification. The XRD of the G-JC, D-JC, B-JC and CNCs are shown in Figure 2. The figure showed that there were diffraction peaks at 2*θ* = 14.8°, 16.4°, 22.2°, and 34.5°, which represented the 1$\bar{1}$0, 110, 200, and 004 planes, respectively, and which are the characteristic peaks of cellulose Iβ [41,43]. All samples maintained similar diffraction patterns, indicating that after chemical purification treatment and further acid hydrolysis, the obtained samples maintained the crystalline structure of cellulose Iβ. At the same time, due to the removal of lignin and hemicellulose in the sample which changed the hydrogen bond between the cellulose chains, there was a slight change in the position of the peak [34].

Since the boundary between the crystalline regions and the amorphous regions of the fiber samples are not clear, the content of the crystalline regions and the amorphous regions cannot be accurately determined. Based on this, we adopted the method described by Segal [44]. Using the difference between the diffraction intensity of the sample on the 200-crystal plane and the diffraction intensity of the amorphous region and its proportion in the diffraction intensity of the 200-crystal plane, the crystallinity index (*CrI*) of the sample was calculated. Usually, we take 2*θ* = 18° as the amorphous phase. The crystallinity of the G-JC, D-JC, B-JC, and CNCs was 49.69%, 54.68%, 66.25%, and 72.36%. Crystallinity was gradually increased with the progress of chemical purification. This was because the initial jujube core powder contains amorphous components such as hemicellulose, lignin, and pectin, which surround the crystalline compounds, resulting in a lower crystallinity. The D-JC and B-JC were the products after the alkali treatment and bleaching treatment, respectively. Because the alkali treatment and bleaching could remove pectin, lignin, hemicellulose, and other components, their crystallinity increased. CNCs were obtained from the B-JC through further sulfuric acid hydrolysis. They had the highest degree of crystallinity. In addition, the crystallinity of the CNCs obtained in this study was higher than that of the CNCs’ crystallinity of 69.99% obtained in Wahib et al.’s study [32]. It was when the hydronium ion from the acid entered the amorphous region in the B-JC which caused the glycosidic bond to be preferentially destroyed. The molecules were rearranged, and the amorphous area was destroyed, thereby increasing the degree of crystallinity [5,45]. Similar results were reported in pineapple peel, and the results showed that the crystallinity index (*CrI*) values of untreated PP, bleached PP, cellulose, and CNCs were 30.72, 42.07, 53.34, and 61.19%, respectively [35]. Due to the effective removal of lignin and hemicellulose by the bleaching treatment and alkali treatment and further acid hydrolysis, the crystallinity of the samples continued to increase.

From the X-ray diffraction pattern, the crystallite size was determined using the Scherrer expression described in Section 4.4.3. By calculating the crystallite size and *d*-spacing (200) of the samples, the *d*-spacing (200) of the samples were basically unchanged. The crystallite size of the G-JC, D-JC, B-JC and CNCs was 2.255, 2.827, 2.957, and 3.147 nm, respectively. Because the glycosidic bonds in the amorphous region are preferentially hydrolyzed and broken during the hydrolysis process, the cellulose molecules are rearranged and the crystallinity is higher. The crystallinity index increases with crystallite size due to the reduction in amorphous regions [46,47].

### 2.4. Thermogravimetric Analysis

**Figure 3:**
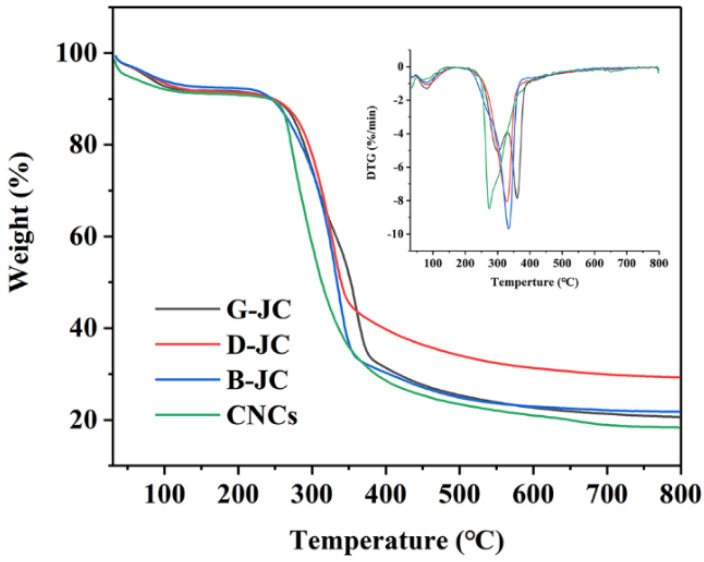
TGA curves of G-JC, D-JC, B-JC and CNCs; the inset image represents the DTG curves for the corresponding thermograms.

Thermogravimetric analysis (TGA) is used to evaluate the thermal properties of materials. Figure 3 shows the TGA and DTG curves of the G-JC, D-JC, B-JC and CNCs in the range of 30 °C–800 °C. The specific results are shown in Table 2. All samples showed three degradation stages. The first stage occurred at 30 °C–150 °C due to the evaporation of intermolecular H-bonded water, free moisture, or degradation of low molecular weight compounds in the sample. The second stage occurred at 210 °C–370 °C, where cellulose chains underwent depolymerization and degradation due to glycosidic bond cleavage. This stage produced and released flammable volatiles through reactions such as dehydration, hydrolysis, oxidation, decarboxylation, and trans-glycosylation [5,39]. The third stage was when the carbon residue was further oxidized and decomposed into low molecular weight components after being heated, such as hydrogen, ethylene, ethane, and other volatile substances [48].

**Figure 4:**
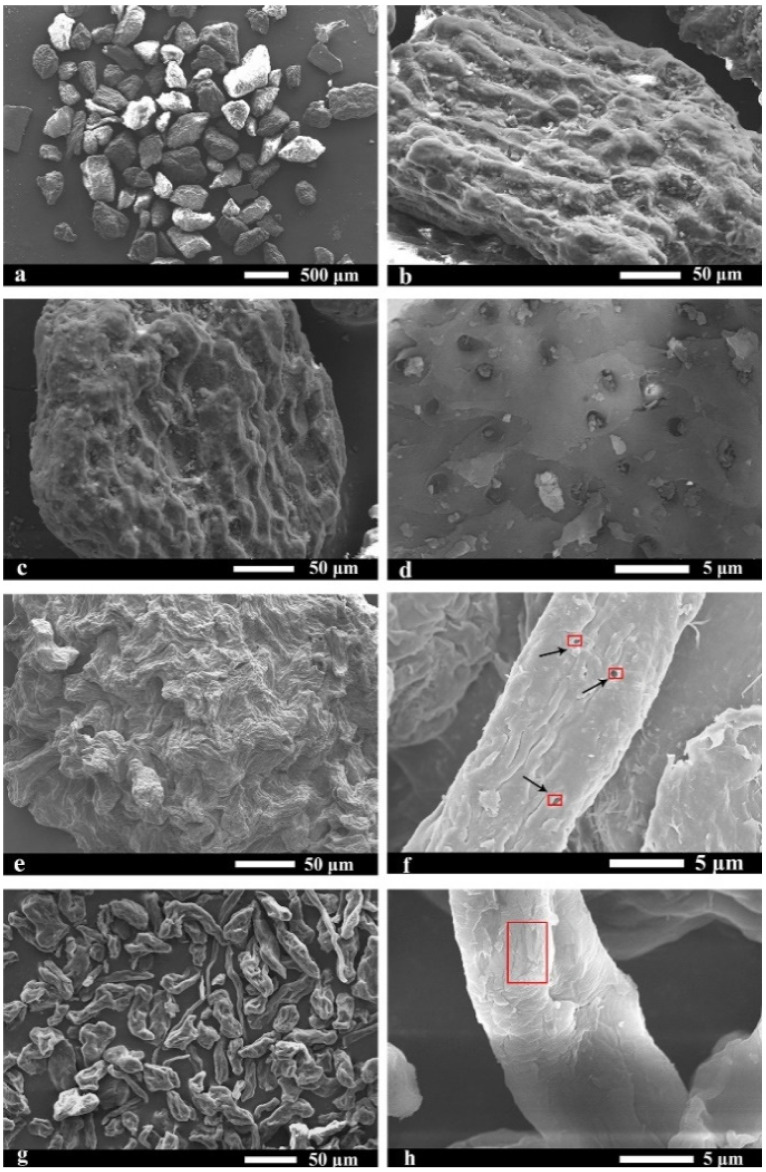
FE-SEM images (**a**,**b**) of G-JC with different magnifications; images (**c**,**d**) of DW-JC with different magnifications; images (**e**,**f**) of D-JC with different magnifications; images (**g**,**h**) of B-JC with different magnifications.

Research had shown that the decomposition temperatures of hemicellulose and cellulose were 250 °C–320 °C and 320 °C–400 °C, respectively, but the decomposition temperature of lignin was more widely in the range of 100 °C–900 °C [39]. It could be obtained that G-JC showed a dominant peak of degradation at 298 °C from DTG in Figure 4, which corresponded to the decomposition of hemicellulose, but there were no corresponding degradation peaks in the D-JC and B-JC, which further proved that the alkaline treatment effectively removed hemicellulose from the jujube core powder (G-JC) and showed consistent results with FT-IR and XRD [39,41,49]. The D-JC and B-JC showed dominant peaks of degradation at 360 °C and 275 °C from DTG in Figure 4, which corresponded to the decomposition of cellulose chains. The decrease in degradation temperature was attributed to the gradual removal of hemicellulose and lignin and the increase in crystallinity [34,50]. In addition, compared with other samples, the Ti value of CNCs was relatively low, which indicated that CNCs had lower thermal stability than the G-JC, D-JC, and B-JC. This was due to the large size and surface area of the CNCs, which made them more easily exposed during heating and led to an acceleration in the thermal degradation rate and, ultimately, to a decrease in their stability [39,48,51].

### 2.5. Morphological Analysis

The field emission scanning electron microscope (FE-SEM) can observe the structural changes before and after the treatment of the jujube core powder fibers. Figure 4 shows FE-SEM images of the grated jujube cores: G-JC (Figure 4a,b); dewaxed jujube cores: DW-JC (Figure 4c,d); delignified jujube cores: D-JC (Figure 4e,f); and jujube core cellulose: B-JC (Figure 4g,h). It is obvious that the morphology of the samples before and after processing had changed significantly. Due to the presence of a large amount of lignin, hemicellulose, pectin, wax, and other ingredients, the irregular block surface of G-JC in Figure 4a,b was dense and smooth, but there were many granular substances. After the Soxhlet extraction, the wax and other substances on the surface of the jujube core powder (G-JC) were removed; the obtained DW-JC in Figure 4c,d was basically the same as the G-JC morphology, except for the reduction of surface particles [5,39].

Lignin, hemicellulose, pectin, and other components were removed after further alkali treatment and bleaching treatment. Figure 4e,f shows that the surface particles of the D-JC completely disappeared and some small pores appeared, which facilitated the penetration of NaClO_2_, resulting in a pure cellulose B-JC as shown in Figure 4g,h. Compared with other samples, the diameter of the B-JC was significantly reduced in Figure 4h, which further indicated the removal of non-fiber components, thereby separating individual fibers [46]. Figure 4h shows that the cellulose fiber surface was relatively rough and cracks appeared. This was due to the penetration of a high-concentration NaOH solution into the fibrils, causing fiber expansion and structural damage [52]. These results support the results of the FT-IR, XRD, and TGA on lignin and hemicellulose removal.

**Figure 5:**
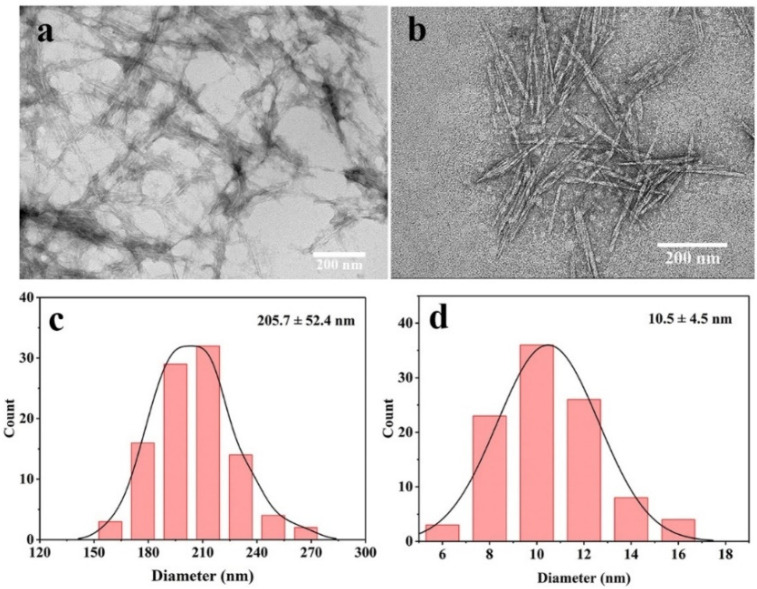
TEM images of: (**a**) CNCs at 30,000× magnifications; (**b**) CNCs at 40,000× magnifications; (**c**) length distribution of obtained CNCs; (**d**) diameter distribution of obtained CNCs.

A transmission electron microscope (TEM) was used to observe the morphology of CNCs. Figure 5 shows the TEM image of CNCs obtained by acid hydrolysis. The TEM results in Figure 5a,b show that the CNCs produced by acid hydrolysis showed a rod-like structure, which was consistent with the CNCs produced by other sources before [34,39,46]. CNCs have slight agglomeration, as is shown from Figure 5a, due to the presence of intermolecular and intramolecular hydrogen bonds and the high specific surface area of CNCs [53]. A total of 100 individual CNCs were selected to fit the diameter in Figure 5c and length in Figure 5d by Nano Measurer software. Statistical data showed that the CNCs had a length of 205.7 ± 52.4 nm, diameter of 10.5 ± 4.5 nm, and length-to-diameter ratio of 20.5. The aspect ratio is an important factor in determining the performance of CNCs. The high aspect ratio makes CNCs have stronger rigidity, which can increase the mechanical strength of the bio-composite [54]. In the composite material, CNCs have strong interactions with other substances through the hydrogen bonding force and through their own abundant hydroxyl groups that maintain the percolation network. For example, Peresin et al. [55] studied CNCs as reinforcements to enhance the mechanical properties of polyvinyl alcohol fiber nanocomposites. The CNCs maintained the permeable network through hydrogen bonding and there was an efficient stress transfer between CNCs and polyvinyl alcohol, resulting in a significant increase in the elastic modulus of the obtained nanocomposites.

**Figure 6:**
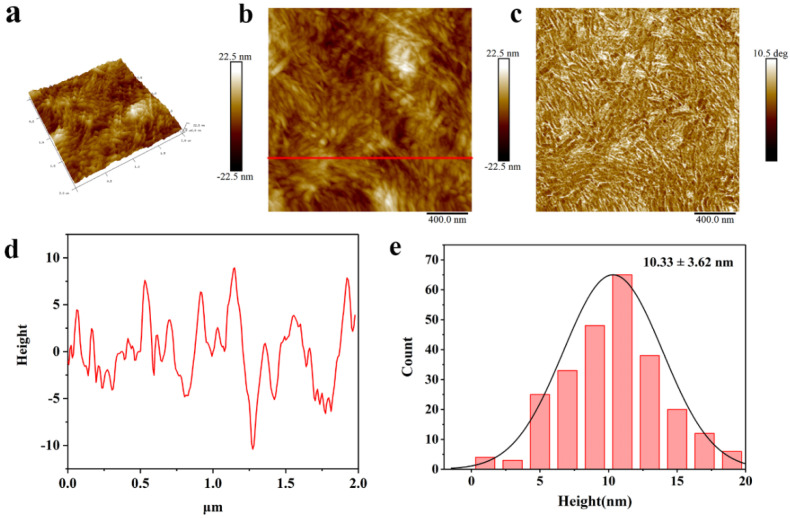
AFM images of CNCs: (**a**) 3D-view image; (**b**) height image; (**c**) phase image; (**d**) height profiles at line positions; (**e**) height distributions.

An atomic force microscope (AFM) was used to analyze the morphology and height of the CNCs. Figure 6 shows the surface topography and height distribution image of CNCs scanned by the AFM. In Figure 6a, the AFM image of CNCs shows needle-like crystals, like the results reported by Kassab et al. [56]. In Figure 6d,e, the height of the CNCs were analyzed; the crystal heights of the CNCs were mainly distributed in the range of 5–15 nm and the average height was 10.33 ± 3.62 nm, which was consistent with the diameter of CNCs shown by TEM as 10.5 ± 4.5 nm. These results were basically the same. The AFM chart also shows that CNCs have a very high specific surface area, which has been shown to be conducive to enhancing the interaction between composite materials [57]. Due to the abundance of hydroxyl groups on the surface of CNCs, they are suitable for many types of surface functionalization. For example, by chemical grafting, various functional molecules, such as fluorescent molecules, DNA, etc., can also be attached to the surface of a CNC, which can be widely used in smart materials [19,58]. In addition, they are also well used in the field of biomedicine. The use of CNC-enhanced nanocomposite membranes in dialysis can combine active ion exchange and passive ultrafiltration to achieve better dialysis effects [59].

### 2.6. Particle Size and Zeta (ζ) Potential Measurements

**Figure 7:**
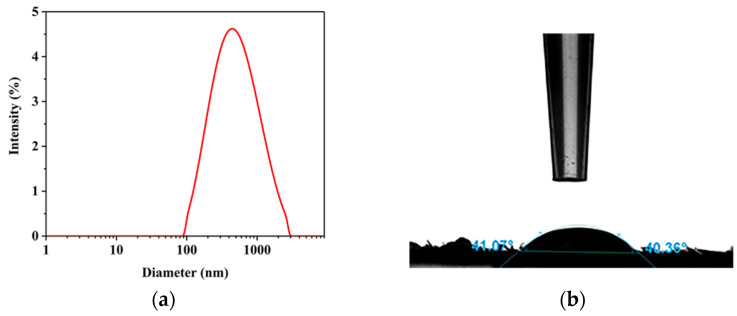
Particle size distribution (**a**) of CNCs obtained and the water contact angle (WCA) (**b**) of CNC powder.

The particle size distribution of the CNCs is shown in Figure 7a. The figure clearly shows that the particle size of the CNCs was mostly distributed in the range of 100–1000 nm. Through the statistical analysis of the instrument’s own software, the CNCs had an average particle size of 382 nm and a PDI of 0.321. This light scattering technique cannot measure the size of a particle accurately and precisely, but it measured the length and diameter dimension of the particles thoroughly. According to the TEM observation, the length of a single CNC was about 200 nm, and the CNCs were aggregated to form larger aggregates as shown in Figure 5a. The average particle size was larger than the particle size obtained by TEM. This was due to the presence of intermolecular hydrogen bonding forces and the formation of aggregates by CNCs appearing in suspension, which exhibited larger hydraulic radii due to light scattering.

The zeta (ζ) potential is an important parameter for analyzing the stability of the CNCs’ aqueous suspension. Zeta potential measurements investigate the degree of electrostatic repulsion between adjacent, similarly charged particles in dispersions and provide broad insights into the dispersion, aggregation, or flocculation of nanoparticles, such as CNCs, in colloidal systems. Studies have shown that solution systems have better colloidal stability when their zeta potentials are below −30 mV or above +30 mV. The effective electrostatic repulsion between the charges on the limits of the particle represent their mutual coagulation or aggregation [41]. This study showed that the zeta potential of the CNCs’ suspension was −23.72 ± 1.7 mV. It was due to the grafting of negatively charged SO_3_^−^ groups on CNCs during acid hydrolysis which made the CNCs’ suspension appear to have negative electrostatic repulsion [60,61]. However, the potential value of the suspension is slightly higher than −30 mV, indicating slight aggregation and precipitation of CNCs in the suspension due to the low electrostatic repulsion between suspended particles. Both particle size and zeta (ζ) potential measurements indicated that the CNCs exhibited slight agglomeration in the suspension system, which was consistent with TEM results (Figure 5a).

### 2.7. Water Contact Angle (WCA)

The water contact angle is used to analyze the surface wetting ability of CNC powder. The static WCA of the film compressed by CNC powder was collected as shown in Figure 7b, and the static WCA of the CNCs’ film was 40.36°. The results showed that the CNCs’ film had strong hydrophilicity. Bruel et al. [62] reported similar results. The static WCA of the film prepared from compressed CNC particles was 45°. This may be due to a large number of hydrogen bonds between or in the molecules of CNCs, which makes them sensitive to water molecule adsorption; thus, the film prepared by compressed CNC particles exhibits strong hydrophilicity [63].

## 3. Discussion

CNCs were successfully separated from jujube cores through dewaxing, alkali treatment, bleaching, and acid hydrolysis. Through the analysis of the chemical compositions of the fibers, it was shown that the purity of the fibers greatly improved from the original 40.48 ± 1.37% to 80.47 ± 2.74%, as the chemical treatment effectively removed the non-fiber components in the jujube cores. The results of FT-IR and XRD further confirmed the efficient removal of lignin and hemicellulose through different treatment stages, which were consistent with the microscopic morphology. The crystal structure of cellulose Iβ was preserved by the CNCs and the crystallinity was 72.36%. The TGA results showed that the thermal degradation temperatures of the fibers were getting lower in different processing stages, which was attributed to the gradual removal of hemicellulose and lignin and the increase in crystallinity, but the CNCs still showed excellent thermal stability (>200 °C). The FE-SEM, TEM and AFM were used to analyze the changes in the microscopic morphology of the fibers at different processing stages, and the results showed that the morphology of the treated fibers changed dramatically and the particle size of the treated fibers decreased gradually due to the removal of non-fiber components. The obtained CNCs were rod-shaped, with a length of 205.7 ± 52.4 nm and a diameter of 10.5 ± 4.5 nm, showing a high aspect ratio (20.5) and a large surface area. The particle size obtained by nanoparticle size analysis was slightly larger than the result of TEM because the CNCs showed slight agglomeration in the aqueous suspension, which was consistent with the zeta (ζ) potential (−23.72 ± 1.7 mV). Furthermore, the results of the WCA showed that the films prepared by CNC powder exhibit strong hydrophilicity due to many hydrogen bonds within, or between, the cellulose molecules. Therefore, the jujube cores, as production waste, are an interesting source of CNCs, and the obtained CNCs have great potential as reinforcing materials for nanocomposites.

## 4. Materials and Methods

### 4.1. Chemicals and Raw Materials

Jujube cores were provided by Xinjiang Yeheyuan Fruit Industry Co., Ltd. (Kashgar, China). The dirt on the surface of the jujube cores was removed with clean water and the material was then dried in an oven at 60 °C for 24 h. Sodium hydroxide was purchased from Tianjin Yongsheng Chemical Co., Ltd. (Tianjin, China), sodium chlorite (80%, *w*/*w*) was purchased from Shanghai Macklin Biochemical Co., Ltd. (Shanghai, China), and sulfuric acid (98%, *w*/*w*) was purchased from Beijing Chemical Works (Beijing, China). Dialysis membranes were also used (molecular weight cut-off: 8000–14,000 Da). Other chemical reagents used in this study were of analytical grade and used without further purification.

### 4.2. Fractionation and Purification of Cellulose from Jujube Cores

**Figure 8:**
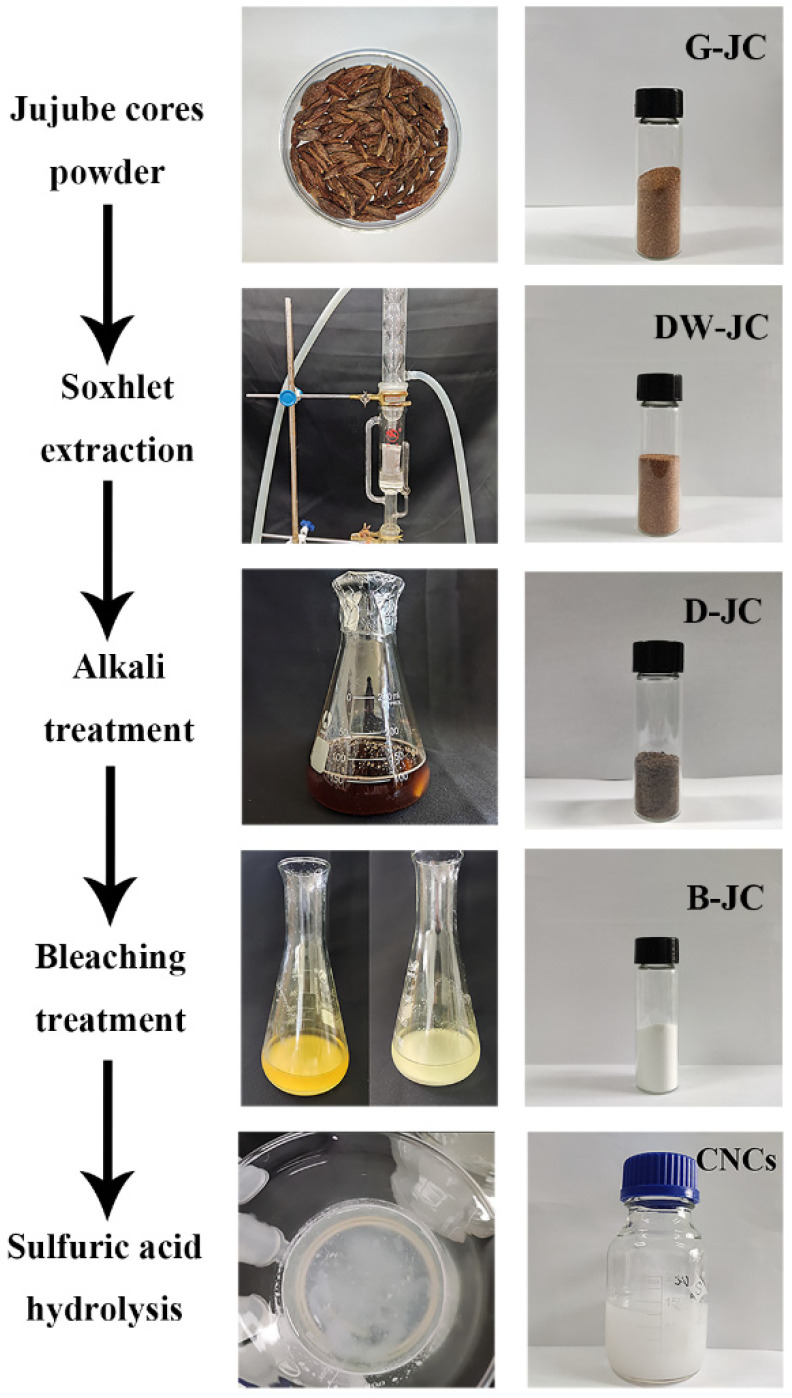
Process for the isolation of CNCs from jujube cores. G-JC: grinded jujube cores; DW-JC: dewaxed jujube cores; D-JC: delignified jujube cores; B-JC: jujube core cellulose; CNCs: cellulose nanocrystals.

The procedure of fractional distillation, purification, and extraction of cellulose from jujube core powder refers to the method of Abu-Thabit et al. [5] with appropriate modifications, as shown in Figure 8. The dried jujube core was ground into powder (G-JC, particle size ≤ 0.25 mm). Before alkali treatment, Soxhlet extraction was used in the dewaxing process. The wax was removed by refluxing with a mixed solvent (ethanol:benzene = 1:2) for 8 h at 85 °C to obtain dewaxed jujube cores (DW-JC). The amount of 500 mL of 10% NaOH was added to 25 g of DW-JC, shook in a water bath at 90 °C for 4 h, filtered immediately to remove the dark brown liquid, which was rich in lignin, and then washed continuously with distilled water until the alkaline filtrate became neutral (pH ≈ 7); delignified jujube cores (D-JC) were obtained after drying. An amount of 250 mL of 10% NaOH was added to the D-JC (10 g), then the pH ≈ 4 with glacial acetic acid was adjusted and shook for 1 h in a water bath at 80 °C for bleaching. The mixture, cooled to room temperature, was filtered through No. 42 Whatman paper and washed with distilled water until the filtrate became neutral. The bleaching process was repeated twice. After drying the bleached jujube cores at 45 °C for 24 h, they were pulverized to obtain jujube core cellulose (B-JC) and stored in a vacuum desiccator to prepare CNCs. The yield of jujube core cellulose was 42.3%.

### 4.3. Preparation of Cellulose Nanocrystals

Cellulose nanocrystals (CNCs) were obtained by sulfuric acid hydrolysis of the B-JC, referring to the method of Dai et al. [35] with some modifications. An amount of 5 g of B-JC was mixed with 100 mL of 63.5% H_2_SO_4_ with constant stirring at 45 °C for 40 min, and the reaction was stopped with deionized ice water 10 times after the reaction was finished. Residual sulfuric acid was removed by centrifugation at 8000 rpm for 10 min. The centrifugation process of multiple washes was repeated until the upper liquid became cloudy. The upper turbid liquid was collected and dialyzed against the deionized water for 5 days using a dialysis membrane (8000–14,000 Da, Biotopped MD44) to collect the resulting suspension, the CNCs, which were stored at 4 °C for further analysis.

### 4.4. Characterizations

#### 4.4.1. Chemical Composition

The contents of holocellulose, lignin, and α-cellulose in the G-JC and B-JC was determined by the Technical Association of the Pulp and Paper Industry (TAPPI) standards: T 19 cm-54, T 222 cm-99, and T 203 cm-99, respectively. The hemicellulose content was determined from the value of holocellulose and α-cellulose contents. The extractives and ash content of the G-DC was determined by TAPPI standards: T 204 cm-97 and T 211 om-07. All experiments were performed three times, and the average values were reported as the results.

#### 4.4.2. Fourier Transform Infrared Spectroscopy (FT-IR) Analysis

The resulting dried powder was subjected to infrared spectroscopy using the Bruker Vertex 70v. Prior to analysis, the samples were ground together with KBr, mixed well to the point of homogeneity, and pressed into light transparent flakes. In transmission mode, the spectral signals of the samples at 400–4000 cm^−1^ were recorded and the obtained spectra were smoothed with the software OMNIC 8.2.0.387 (Thermo Fisher Scientific Inc., Waltham, MA, USA).

#### 4.4.3. X-ray Diffraction (XRD) Analysis

The structural changes of the samples were characterized by an X-ray diffractometer (Ultima IV, Rigaku, Tokyo, Japan) using Cu-Kα radiation generated at an operating voltage of 40 kV and a current of 150 mA. Samples were incubated at the 2*θ* range of 5–90° with a step size of 0.02° and the test was performed at a rate of 4 °/min. The resulting diffraction spectra were smoothed and analyzed. The crystallinity index (*CrI*) of the samples was calculated from the following Equation (1) [44]:

(1) $$CrI\left(\%\right)=\frac{I_{200}-I_{am}}{I_{200}}\times 100$$

where *I*_200_ is the maximum intensity of the (200) diffraction at the 2*θ* value of about 22.2°, whereas *I_am_* is the intensity diffraction at the 2*θ* value of around 18°.

The crystallite size perpendicular to the lattice plane, the (200) plane of the samples, was calculated from the Scherrer equation, according to Equation (2), and the *d*-spacing (200) of the samples was calculated by using Bragg’s equation, according to Equation (3):

(2) $$L=\frac{k\lambda }{\beta \cos\theta }$$

where k = 0.9 is the correction factor, λ (1.54056 Å) is the wavelength of the X-ray radiation, β is the FWHM of the diffraction peak in radians, and θ is the diffraction angle of the peak.

(3) 2dsinθ=nλ

#### 4.4.4. Thermogravimetric Analysis (TGA)

Thermogravimetric analysis (STA 449F5, NETZSCH, Bavarian Asia, Germany) was used to study the thermal stability of the G-JC, D-JC, B-JC, and CNCs. The samples (about 5 mg) were placed in the Al_2_O_3_ pots, and the samples were heated from 30 °C to 800 °C at a heating rate of 10 °C/min. All measurements were performed under a nitrogen atmosphere with a gas flow rate of 40 mL/min.

#### 4.4.5. Field Emission Scanning Electron Microscopy (FE-SEM)

The field emission scanning electron microscope (SU 8010, HITACHI, Tokyo, Japan) was used to observe the morphology of samples in different chemical treatment stages (G-JC, DW-JC, D-JC, and B-JC) under an accelerating voltage of 5 kV, referring to Prasanna et al.’s [39] research methods. Before observation, the samples were dried at 60 °C for 12 h, fixed on short aluminum stubs, and all samples were plated with gold using a vacuum sputter coater (SD-900, Boyuan Micro Nano, Beijing, China).

#### 4.4.6. Transmission Electron Microscopy (TEM)

The transmission electron microscope (HT 7700, HITACHI, Tokyo, Japan) was used to analyze the shape and size of CNCs. A drop of 0.05 wt.% of the CNCs’ aqueous suspension was deposited on the carbon film supported by the copper grid (300 mesh), stood for 2 min, and then the CNCs were negatively stained with a 1% phosphotungstic acid solution for 2 min. The excess staining solution was then blotted with filter paper and dried under infrared light. The TEM observation was performed under an acceleration voltage of 100 kV, and the diameter and width of 100 individual CNCs in the resulting image area were measured using Nano Measurer software.

#### 4.4.7. Atomic Force Microscopy (AFM)

An atomic force microscope (Multimode 8, Bruker, Karlsruhe, Germany) was used to measure the surface of the CNCs. In the tapping mode, the phase diagram and the height diagram of the area of 2.0 µm × 2.0 µm was obtained at the same time with a resolution of 256 × 256 points. Before analysis, a drop of 0.01 wt.% of CNCs’ suspension was dropped on a clean silicon wafer surface and dried at room temperature for 12 h.

#### 4.4.8. Particle Size and Zeta Potential Measurements

The particle size and potential of the CNCs were analyzed with a nanoparticle zeta (ζ) potential analyzer (NanoPLUS-3, Micromeritics, Norcross, GA, USA). The suspension of CNCs was diluted to 0.05 wt.% to obtain the statistical distribution of the CNCs’ particle size. The zeta potential of the CNCs was obtained by measuring the mobility of the particles that underwent electrophoresis in each suspension, and then converting it into a zeta potential value. All tests were repeated 3 times.

#### 4.4.9. Water Contact Angle (WCA)

The water contact angle of the CNCs was measured by the fixed drop method using a contact angle meter (Theta Flex, BIOLIN, Gothenburg, Sweden). The CNC powder, obtained by freeze-drying, was pressed into a sheet, a drop of deionized water (5 µL) was dropped on it with a syringe, and the photograph was taken immediately. Each sample was measured eight times.

## Figures and Tables

**Table 2:** Onset temperature (T_On_), degradation temperature at max weight loss (T_max_), weight loss (W_L_), and char yield for G-JC, D-JC, B-JC, and CNCs evaluated from TG and DTG curves.

Sample	Step 1 (Evaporation of Water)			Step 2 (Degradation of Cellulose Chain)			Step 3 (Degradation of Carbonic Residue)			Char Yield (%)
	T_On_ (°C)	T_max_ (°C)	W_L_ (%)	T_On_ (°C)	T_max_ (°C)	W_L_ (%)	T_On_ (°C)	T_max_ (°C)	W_L_ (%)	
G-JC	30	79	8.13	222	298	30.91	330	360	40.34	20.61
D-JC	30	80	8.48	221	328	62.25	352	-	-	29.27
B-JC	30	80	7.56	213	333	70.67	359	-	-	21.76
CNCs	30	72	8.03	209	275	72.84	365	-	-	18.28

## Data Availability

The data presented in this study are available on request from the corresponding author.
